# Correction to “Deep Gray Matters Iron Deposition Is Positively Associated With White Matter Hyperintensity in Hypertension”

**DOI:** 10.1111/jch.70253

**Published:** 2026-04-11

Y. Su, W. Wu, Z. Qin, et al., Deep Gray Matters Iron Deposition Is Positively Associated With White Matter Hyperintensity in Hypertension. *Journal of Clinical Hypertension* 25, no. 8 (2023): 768–777, https://doi.org/10.1111/jch.14694.

In Figure 5 (the elderly subgroup, age > 61 years), the first scatter plot in the published version mistakenly used the corresponding plot from Figure 3. All parts in Figure 5 have now been corrected, and the legend remains unchanged. After thorough verification, it has been confirmed that this figure misplacement does not affect the statistical results or the final conclusions of the study, and is purely an operational error during the figure integration stage. To avoid a misunderstanding among readers, the revised version of Figure 5 is provided below.

**FIGURE 5:**
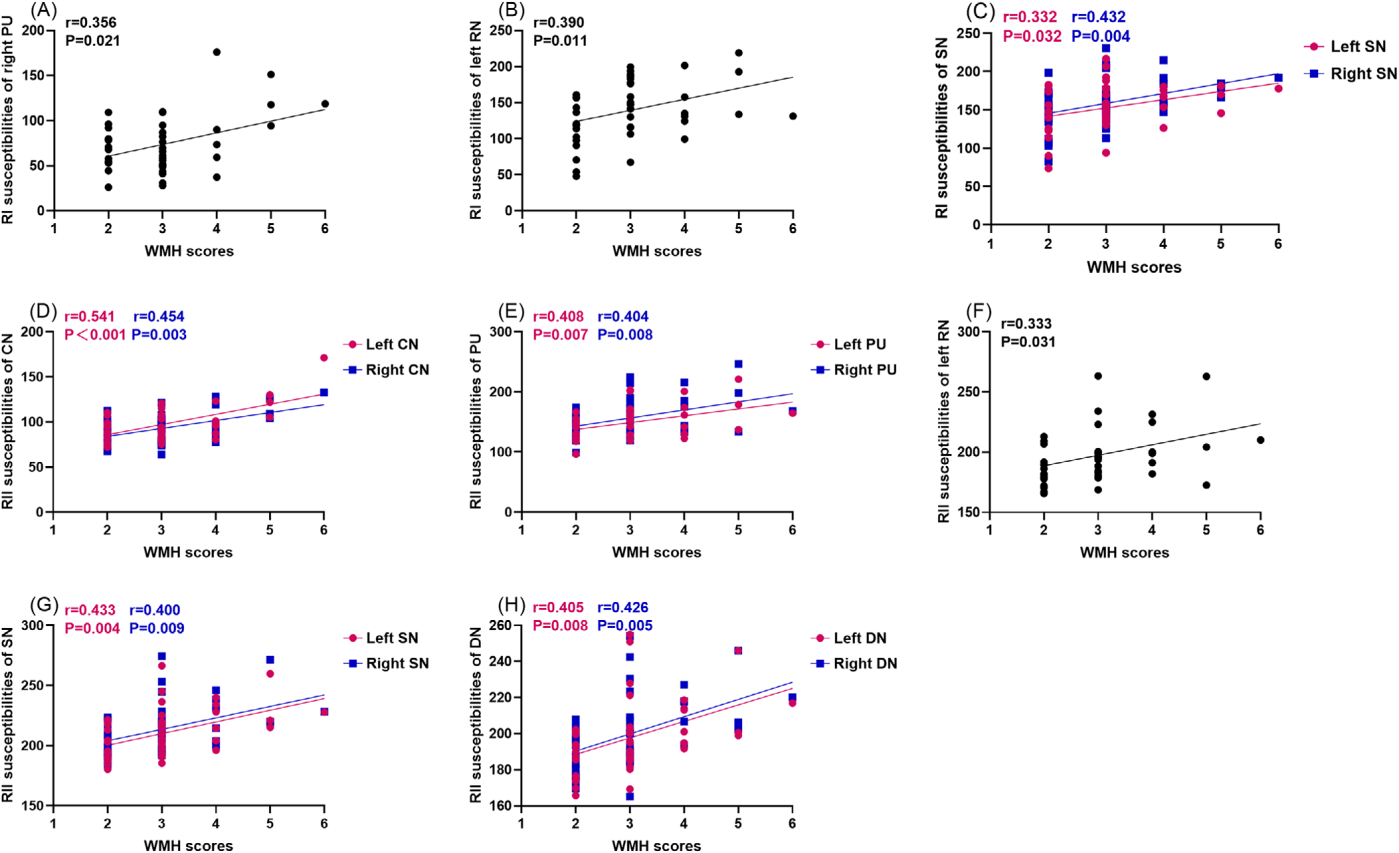
Diagram showing the partial correlation analysis between deep gray matters susceptibilities and WMH, scores in the older (>61 years old) group. (A–C) Correlation between RI susceptibilities (right PU, left RN, and bilateral SN) and WMH scores. (D–H) Correlation between RII susceptibilities (bilateral CN, PU, SN, DN, and, left RN) and WMH scores.

We apologize for this error.

